# The beetles (Insecta, Coleoptera) of the southwest of Primorsky Krai, Russian Far East

**DOI:** 10.3897/BDJ.10.e97992

**Published:** 2022-12-30

**Authors:** Kirill V Makarov, Yuri N Sundukov

**Affiliations:** 1 Moscow State Pedagogical University, Moscow, Russia Moscow State Pedagogical University Moscow Russia; 2 Federal Scientific Center of the East Asia Terrestrial Biodiversity Far Eastern Branch of the Russian Academy of Sciences, Vladivostok, Russia Federal Scientific Center of the East Asia Terrestrial Biodiversity Far Eastern Branch of the Russian Academy of Sciences Vladivostok Russia

**Keywords:** Coleoptera, fauna, distribution, southwest of Primorsky Krai, Russian Far East

## Abstract

**Background:**

The article is based on the results of the authors' field studies on the fauna of Coleoptera in the southwest of Primorsky Krai, conducted in 1990, 1999, 2015 and 2017–2022. The collection of material was carried out in more than 150 geographical points in the territories of the Khasansky, Nadezhdensky and Ussuriysky raion of the Primorsky Krai of Russia. In addition, small collections materials stored at the Federal Scientific Center of East Asia Terrestrial Biodiversity FEB RAS (Vladivostok, Russia) and Moscow Pedagogical State University (Moscow, Russia) were studied. This is the first generalised list of beetles for south-western Primorye and protected natural areas. A total of 13274 beetles belonging to 629 species from 311 genera and 44 families were studied. In addition to our own collections, the sample includes literature data on 10008 specimens belonging to 355 species from 142 genera and 16 families.

**New information:**

This is the first dataset that provides data on the taxonomic composition and geographic distribution with precise coordinates for 47 families of Coleoptera in the southwest of Primorsky Krai, Russia.

## Introduction

The beetle fauna (Insecta, Coleoptera) of the southwest of Primorsky Krai is one of the best studied in the Russian Far East (Lehr 1989, Lehr 1992, Lehr 1996 etc.). However, information on the distribution of most species is very scarce or completely absent. The main goal of our work was to identify the taxonomic diversity of beetles (Coleoptera) and their distribution in the southwest of Primorsky Krai. The most intensive field research was conducted in 2017-2022 and the data were converted to Darwin Core format in 2022.

## Sampling methods

### Study extent

The data are based on material collected by the authors during nine field expeditions to the southwest of Primorsky Krai. During the period of the most intensive studies in 2017–2022, route and stationary studies covered the territory from the village of Khasanskoye in the south to Ussuriysk in the north (Fig. 1). During this period, the collection of beetles was carried out in more than 150 geographical points in the territories of the Khasansky, Nadezhdensky and Ussuriysky raion of Primorsky Krai. As a result, 13,274 Coleoptera specimens belonging to 629 species from 311 genera and 44 families were collected and identified. Processing of the publications cited here made it possible to include in the sample data on 10008 specimens belonging to 355 species from 142 genera and 16 families (Lafer and Zolotarenko (1971), Lafer (1973), Hieke (1976), Lafer (1976), Lafer (1978), Lafer (1984), Kataev (1989), Kataev and Wrase (1995), Moraveč and Wrase (1997), Moraveč and Wrase (1995), Fedorenko (1996), Kurbatov (1993), Ito (1996), Obydov (1996), Zamotajlov (1996), Obydov (1999), Solodovnikov (1999), Lafer (2004), Zamotajlov (2005), Lafer (2005), Bezborodov et al. (2008), Dudko (2006), Lafer and Kataev (2008), Melnik (2009), Miroshnikov (2006), Ruzzier and Kovalev (2016), Gus'kova (2012), Jaeger (2013), Bezborodov (2014), Jendek and Grebennikov (2011), Bezborodov (2015), Bezborodov (2016a), Bezborodov (2016b), Veselova (2011), Polilov (2008), Valainis (2010), Obydov (2009), Ryvkin (2011a), Ryvkin (2011b), Sazhnev (2018), Bezborodov (2019), Schmidt and Liebherr (2009), Guéorguiev and Liang (2020), Lafer and Budilov (2015), Makarov et al. (2019), Sergeev (2019), Sergeev (2015), Shabalin (2013), Sundukov and Makarov (2021)). The complete sample includes 23282 Coleoptera specimens from 862 species, 360 genera and 47 families and the study area covers the Nadezhdensky, Oktyabrsky, Pogranichny, Ussuriysky, Khankaysky, Khasansky and Khorolsky administrative districts of Primorsky Krai (Fig. 1a, Fig. 1b).

### Sampling description

When studying beetles in the southwest of Primorsky Krai, all available methods of collecting were used: manual collection, collection with an exhauster, installation of soil traps, shaking off the crowns of trees and shrubs, catching by electric lamps, night collection with a flashlight, “trampling” of vegetation in wet and swampy biotopes, pouring water in near-water areas, sifting the litter with an entomological sieve, “mowing” the grass with an entomological net and catching with window traps (Chapman and Kinghorn 1955, Hardwick 1968, Stewart and Lam 1968, Gillies 1969, Vanhercke et al. 1981, Kryzhanovskij 1983, Schauff 1986, Spence and Niemelä 1994, Dunaev 1997, Yahiro and Yano 1997, Skvarla et al. 2014).

### Quality control

All collected specimens of the families Agyrtidae, Anobiidae, Carabidae, Cerambycidae, Coccinellidae, Cucujidae, Decliniidae, Disteniidae, Elmidae, Endomychidae, Erotylidae, Geotrupidae, Helotidae, Kateretidae, Lucanidae, Melandryidae, Meloidae, Monotomidae, Mordellidae, Mycetophagidae, Ochodaeidae, Pyrochroidae, Rhysodidae, Silphidae and Tenebrionidae have been identified by the authors of this resource. The species of other families are partially or completely determined by specialists: A.A. Gusakov (Bolboceratidae, Scarabaeidae, Trogidae), S.V. Kazantsev (Cantharidae, Lycidae), A.G. Kirejtshuk (Nitidulidae), B.M. Korotyaev, I.A. Zabaluev (Curculionidae), S.V. Kurbatov (Pselaphidae, Scydmaenidae), I.V. Melnik (Buprestidae, Cleridae, Trogossitidae), Yu.E. Mikhailov, M.E. Sergeev (Chrysomelidae, Megalopodidae), A.A. Petrov (Scolytidae), A.A. Polilov (Ptiliidae), A.A. Prokin (Elmidae), A.S. Sazhnev (Heteroceridae), S.V. Saluk (Latridiidae), O.I. Semionenkov, A.S. Tokareva (Staphylinidae) and V.K. Zinchenko (Leiodidae).

Systematics and names of taxa are given in accordance with the Catalogue of Palaearctic Coleoptera (Löbl and Smetana 2006, Löbl and Smetana 2007, Löbl and Smetana 2010, Löbl and Smetana 2011, Löbl and Smetana 2013, Löbl and Löbl 2015, Löbl and Löbl 2017, Löbl and Löbl 2020).

## Geographic coverage

### Description

The southwest of Primorsky Krai is located in the extreme southeast of mainland Russia. According to the physical-geographical zoning of the south of the Far East, it occupies the western part of the Khanka-Suifun physical-geographical region (Liverovskii and Kolesnikov 1949). Botanically and geographically, the flat part of the region belongs to the forest-steppe zone and the mountainous parts belong to the subzone of liana coniferous-deciduous forests (Liverovskii and Kolesnikov 1949, Petropavlovsky 2004). The presented territory is located at the coordinates 42.29° –45.35° north latitude and 130.39° –132.41° east longitude (Fig. 1a). Its western border runs along the state border of Russia with China and North Korea, the eastern - along the western shore of Lake Khanka, the valleys of the Ilistaya and Razdolnaya rivers and the coast of Peter the Great Bay. The length from north to south is about 350 km, the maximum length from west to east is about 100 km.

According to the nature of the relief in the south-west of Primorsky Krai, two parts are distinguished: the eastern plain and the western mid-mountain. The flat territory in the north is represented by the Khanka lowland, in the centre by the Ussuri lowland and the delta of the Razdolnaya River, in the extreme south - a small alluvial-marine plain on the site of the ancient delta of the Tumannaya River (Nikolskaya 1969). The orographic composition of the mountainous territory includes the Pogranichny Range (the highest point is Mount Kedrovaya, 984 m above sea level) in the north, the Borisov Plateau (Mount Pologaya, 741 m above sea level) in the centre and the Black Mountains Range (Mount Luna, 919 m above sea level) in the south.

The most common vegetation of the plains is meadows, less often steppes (Kurentsova 1962). This vegetation is represented by wet reed grass meadows, moderately moistened and dry grass-grass meadows and grass swamps. Forest areas are fragmentary, occurring on small hills, along riverbeds and on the coastal ridges of Khanka Lake and Posyet Bay. Nemoral and mountain-nemoral oak and polydominant broad-leaved forests predominate in mountainous areas (Petropavlovsky 2004). Within the Borisov Plateau, mountain larch and spruce-fir forests with nemoral herbage are common.

The south-western part of Primorsky Krai is a unique for Russia refugium of warm-temperate East Asian flora and fauna, distinguished by a high level of biodiversity amongst the regions of the Russian Far East. Therefore, a significant part of this territory is part of specially protected natural areas of the federal (“Land of the Leopard” National Park, “Kedrovaya Pad” Nature Reserve, Far Eastern Marine Reserve (Fig. 1b) and western clusters of Khankaysky Nature Reserve) and regional (Nature Reserves Khasansky, Komissarovsky and Poltavsky) values.

The most important feature of the flora of the territory is the high saturation with subtropical and tropical species, which hardly penetrate here into the territory of Russia from adjacent regions of East Asia (Kozhevnikov et al. 2005). For example, south-western Primorye includes 46% of all Far Eastern and 87% of all Primorsky Krai angiosperm species included in the Red Book of the Russian Federation (Anonymous 2008). Moreover, 30% of the rare species of Primorsky Krai are found only in its southwest. The same can be said about gymnosperms (50% of the entire Far East and 80% of Primorsky Krai), lichens (49% and 74%, respectively) or fungi (50% and 50%, respectively) (Anonymous 2008). No less noticeable is the contribution of the southwest of Primorsky Krai to the conservation of animal diversity. For example, this territory is inhabited by 55% of Far Eastern and 100% of protected species of terrestrial mammals of Primorsky Krai (50% is found only here), 67% of Far Eastern and 100% of reptiles of Primorsky Krai (50% is found only here), 60% of Far Eastern and 78% of protected insects of Primorsky Krai and 100% annelids of Far East and Primorsky Krai (Anonymous 2021).

The share of beetles included in the Red Data Book of Russia is also high. In the southwest of Primorsky Krai, 64% of Far Eastern and 90% of rare beetle species of Primorsky Krai are found, 30% of which are found only in this territory (Anonymous 2021). In addition to the species included in the Red Book, a number of taxa have been noted in the southwest of Primorsky Krai that are also distributed in the modern fauna of foreign East Asia, but do not go beyond southwest of Primorsky Krai in their distribution. For example, amongst ground beetles (Carabidae), these include *Amarasilvestrii* Baliani, 1937, *Anisodactyluspunctatipennis* A. Morawitz, 1862, *Bembidioncoreanum* Jedlička, 1946, *Brachinusaeneicostis* Bates, 1883, *Carabusfraterculus* Reitter, 1895, *C.hummelipusongensis* Imura, 1993, *C.jankowskii* (Oberthür, 1883), *C.smaragdinuslosevi* Rapuzzi, 2016, *C.wulffiusi* A. Morawitz, 1862, *Cymindiskuznetzowi* Sundukov, 2001, *Dromiusjureceki* (Jedlička, 1935), *Harpaluschasanensis* Lafer, 1989, *H.farkaci* Kataev et Wrase, 1995, *Mastaxthermarumegorovi* Lafer, 1973, *Nebriakomarovi* Semenov et Znojko, 1928, *Nipponoharpalusdiscrepans* (A. Morawitz, 1862), *Stenaptinusagnatus* (Chaudoir, 1876), *Pogonusitoshimaensis* Habu, 1954, *Pristosiavigil* (Tschitschérine, 1895), *Pterostichuscoruscus* (Tschitschérine, 1895), *P.jungens* (Tschitschérine, 1893), *P.kerzhneri* Lafer, 1983, *P.mukdenensis* Breit, 1933, *P.orientalisnigromontanus* Lafer et Budilov, 2015, *Scaritesterricolapacificus* Bates, 1873, *Tachyuragradata* Bates, 1873 and some others.

It should be noted that the southwest of Primorsky Krai is one of the most populated parts of the Russian Far East (population density is about 10 people/km^2^). A characteristic feature of this part of the region is a rather high degree of environmental transformation. Primary ecosystems here co-exist with secondary ones and a significant part of the forests has been subjected to fires or economic felling. Therefore, the publication of the results of our research seems relevant, as it can serve for the purposes of further monitoring.

### Coordinates

42.29 and 45.35 Latitude; 130.39 and 132.41 Longitude.

## Taxonomic coverage

### Description

Data on 862 species of the order Coleoptera belonging to 360 genera and 47 families are given (Sundukov and Makarov 2022).

### Taxa included

**Table:** 

Rank	Scientific Name	Common Name
order	Coleoptera	Beetles

## Temporal coverage

**Data range:** 1870-6-05 – 2022-6-09.

## Usage licence

### Usage licence

Creative Commons Public Domain Waiver (CC-Zero)

## Data resources

### Data package title

The Coleoptera of the southwest of Primorsky Krai, Russian Far East

### Resource link

https://www.gbif.org/ru/dataset/d90681d8-285b-4682-b2f6-f8929dfe6899

### Alternative identifiers

http://gbif.ru:8080/ipt/resource?r=colswprim&v=1.2

### Number of data sets

1

### Data set 1.

#### Data set name

The Coleoptera of the southwest of Primorsky Territory, Russian Far East

#### Data format

Darwin Core

#### Character set

UTF-8

#### Download URL

https://www.gbif.org/ru/occurrence/download?dataset_key=d90681d8-285b-4682-b2f6-f8929dfe6899

#### Description

The dataset includes the results of the authors' field studies on the fauna of Coleoptera in the southwest of Primorsky Krai, conducted in 1990, 1999, 2015 and 2017–2022. The collection of material was carried out in more than 150 geographical points in the territories of the Khasansky, Nadezhdensky and Ussuriysky raion of the Primorsky Territory of Russia. In addition, small collection materials stored in the Federal Scientific Center of East Asia Terrestrial Biodiversity FEB RAS (Vladivostok, Russia) and Moscow Pedagogical State University (Moscow, Russia) were studied. A total of 13274 beetles belonging to 629 species from 311 genera and 44 families were studied. In addition to our own collections, the sample includes literature data, including information on 10008 specimens belonging to 355 species from 142 genera and 16 families.

**Data set 1.** 

Column label	Column description
basisOfRecord	Preserved Specimen (in all table).
class	Insecta (in all records).
continent	Asia (in all records).
coordinateUncertaintyInMetres	The horizontal distance (in metres) from the given decimalLatitude and decimalLongitude describing the smallest circle containing the whole of the Location.
country	Russia (in all records).
countryCode	Country code, RU in all records.
county	Full, unabbreviated name of the next smaller administrative region than state Province in which the Location occurs.
day	The integer day.
decimalLatitude	The geographic latitude.
decimalLongitude	The geographic longitude.
eventDate	The date or interval during which an Event occurred.
family	Full scientific name of the family in which the taxon is classified.
genus	Generic name.
geodeticDatum	Geodetic datum, WGS84 in all records.
habitat	Category or characteristic of the habitat in which the beetles are collected.
infraspecificEpithet	The name of the lowest or terminal infraspecific epithet of the scientificName, excluding any rank designation.
kingdom	Animalia (in all records).
locality	The specific description of the place.
LocationRemarks	Comments or notes about the Location. Specially protected natural areas of Primorsky Krai under the leadership of the Federal State Budgetary Institution "Land of the Leopard": Far Eastern Marine Reserve, Gamovsky cluster, Kedrovaya Pad Nature Reserve, Land of the Leopard National Park.
month	The integer month.
occurrenceID	An composite identifier for Occurrence: the first 4 letters of the generic epithet, 10 letters from the specific epithet and the ordinal number of the entry for this species.
order	Coleoptera (in all records).
organismQuantity	A number value for the quantity of specimens.
organismQuantityType	The type of quantification system used for the quantity of organism.
phylum	Arthropoda (in all records).
recordedBy	A person, responsible for recording the original Occurrence.
scientificName	The full scientific name, including author and year.
specificEpithet	The name of the first or species epithet of the scientificName.
stateProvince	Primorsky (Maritime) Krai, in all records.
taxonRank	The taxonomic rank of the most specific name in the scientificName (species or subspecies).
verbatimCoordinates	The verbatim original spatial coordinates.
verbatimCoordinateSystem	In all table: degrees, minutes, seconds.
verbatimEventDate	The verbatim original representation of the date information.
verbatimLocality	The original textual description of the place.
year	The four-digit year.

## Figures and Tables

**Figure 1a.**
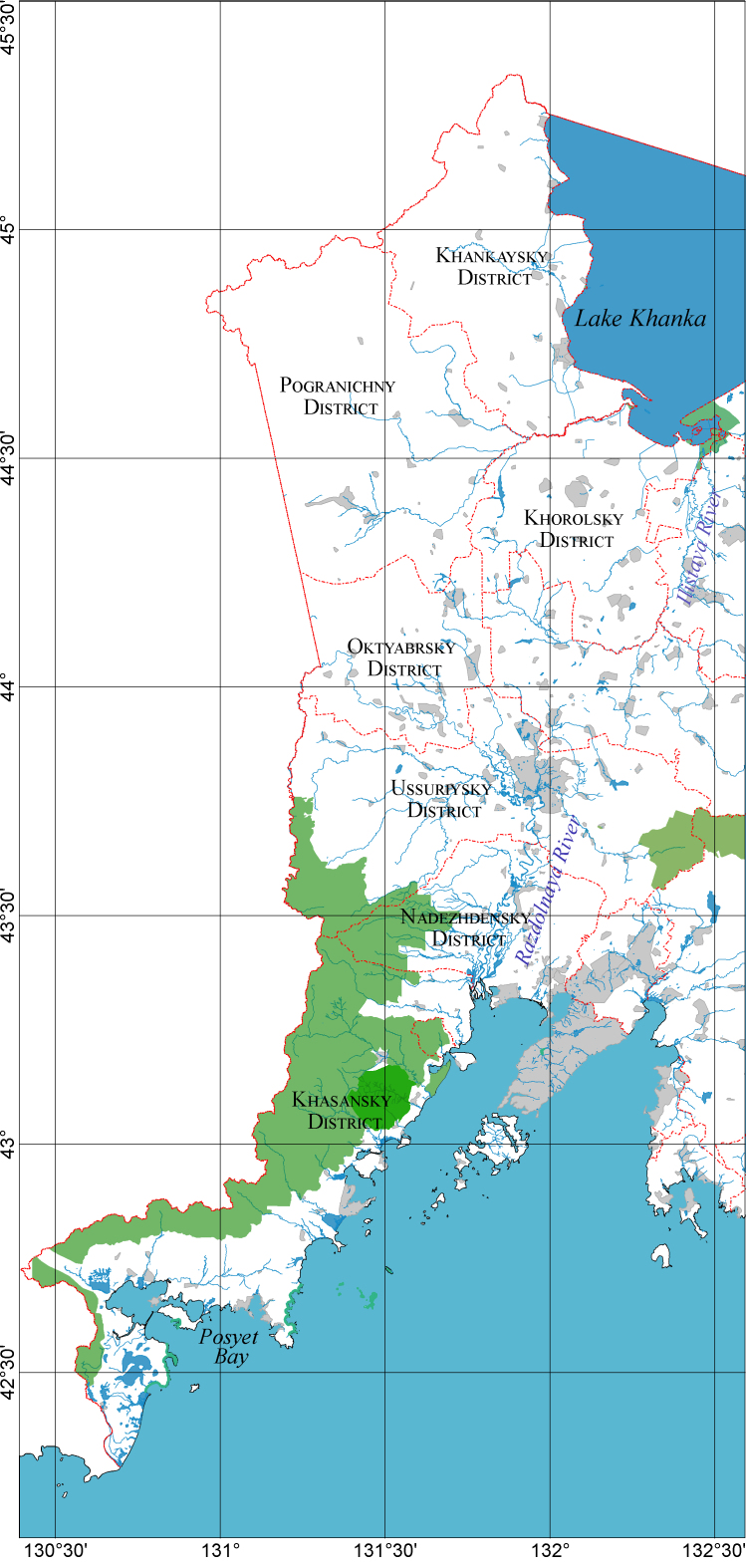
administrative districts

**Figure 1b.**
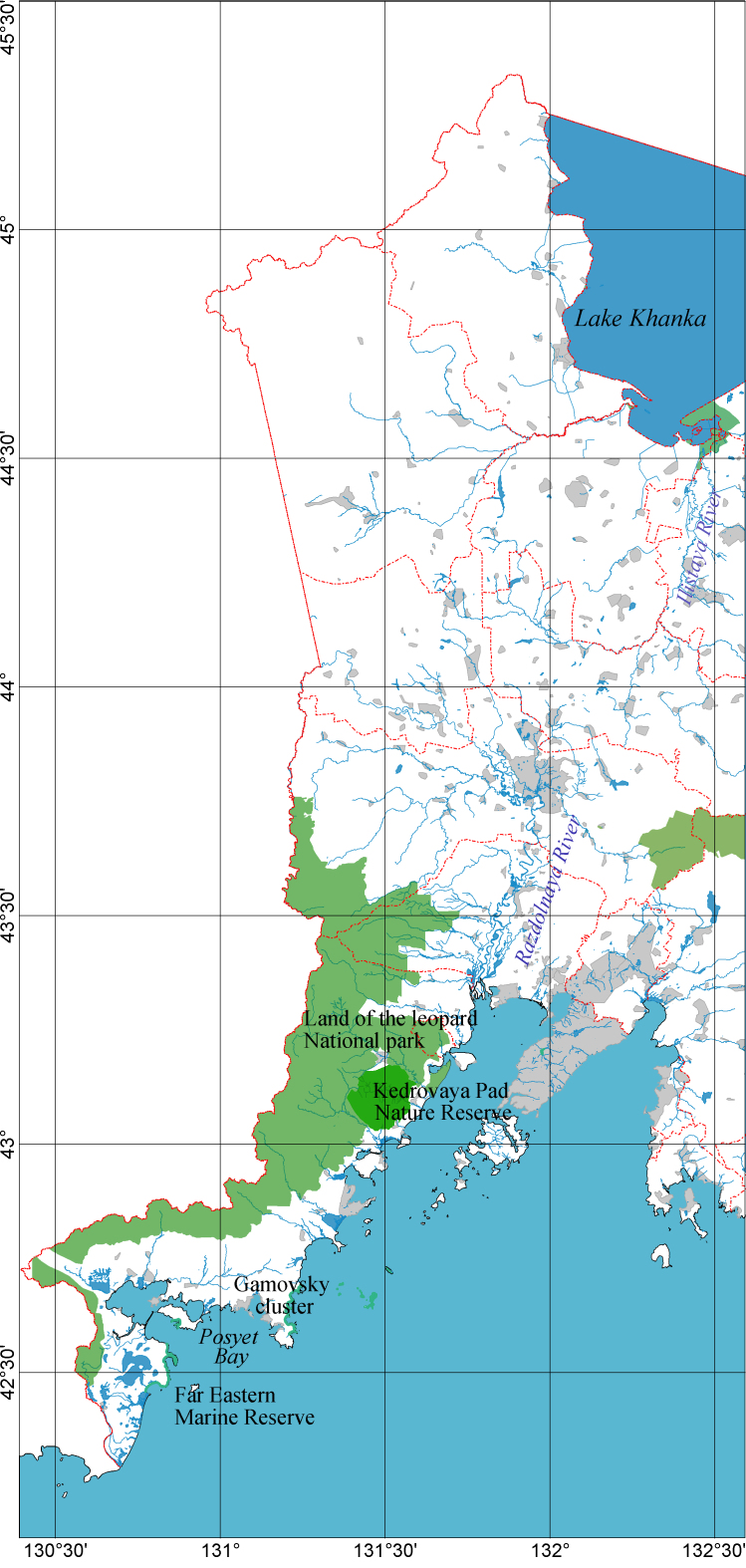
specially protected natural areas.
